# Probing heat generation during tensile plastic deformation of a bulk metallic glass at cryogenic temperature

**DOI:** 10.1038/s41598-018-34681-4

**Published:** 2018-11-05

**Authors:** David D. E. Brennhaugen, Konstantinos Georgarakis, Yoshihiko Yokoyama, Koji S. Nakayama, Lars Arnberg, Ragnhild E. Aune

**Affiliations:** 1 Department of Materials Science and Engineering, NTNU, Norwegian University of Science and Technology, 7491 Trondheim, Norway; 2 School of Aerospace, Transport and Manufacturing, Cranfield University, Cranfield, MK43 0AL UK; 3 Institute for Materials Research, Tohoku University, 2-1-1, Katahira, Sendai, 980-8577 Japan; 4 New Industry Creation Hatchery Center, Tohoku University, 2-1-1, Katahira, Sendai, 980-8577 Japan

## Abstract

Despite significant research efforts, the deformation and failure mechanisms of metallic glasses remain not well understood. In the absence of periodic structure, these materials typically deform in highly localized, thin shear bands at ambient and low temperatures. This process usually leads to an abrupt fracture, hindering their wider use in structural applications. The dynamics and temperature effects on the formation and operation of those shear bands have been the focus of long-standing debate. Here, we use a new experimental approach based on localized boiling of liquid nitrogen by the heat generated in the shear bands to monitor the tensile plastic deformation of a bulk metallic glass submerged in a cryogenic bath. With the “nitrogen bubbles heat sensor”, we could capture the heat dissipation along the primary shear banding plane and follow the dynamics of the shear band operation. The observation of nitrogen boiling on the surface of the deforming metallic glass gives direct evidence of temperature increase in the shear bands, even at cryogenic temperatures. An acceleration in bubble nucleation towards the end of the apparent plastic deformation suggests a change from steady-state to runaway shear and premonitions the fracture, allowing us to resolve the sequence of deformation and failure events.

## Introduction

Metallic glasses are a unique group of amorphous materials typically produced by rapid quenching of some selected alloys. While mechanically very strong, these glasses fracture differently from most other metals in that their deformation is localized to thin bands, intersecting the sample body at ~45°^1^ to the stress axis. The hows and whys of these shear bands are therefore very important questions for improving deformability in metallic glasses and making them practically applicable.

Although amorphous, a metallic glass is not purely homogeneous at the nanometric scale^2^. The structural origin of shear banding in metallic glasses is thought related to the coalescence of low-density-and-strength atomic clusters, called shear transformation zones, under applied stress^3,4^, into ~20 nm^5,6^ thin planes. Viscosity reduction through structural dilation^7^ then causes preferential deformation in these bands, with the final fracture typically leaving traces of completely molten material on the surface in the form of Saffman-Taylor fingering^8,9^. With viscosity and temperature being intimately related, and considering these shear bands are high-friction environments, the role of temperature and heating in the failure of metallic glasses has received a fair amount of attention. Significant work has focused on the effect of ambient temperature, since cryogenic temperatures have an apparent stabilizing effect on plastic deformation^10–13^, as well as on the heating of the shear band itself^7,14–20^. Various methods applied to disentangle structural and caloric effects include coating the metallic glass sample with fusible coatings with known melting points^15,16^, high frequency infrared thermography^21,22^, back-calculations from estimated viscosity in shear bands^23^ and theoretical approaches based on release of elastically stored energy in the sample^19,24^ and heat release from shear deformation^25^.

Lewandowski and Greer studied shear band temperature using their novel fusible coating technique, where they coated samples in tin before bend testing, and observed local melting along shear bands^15^. They confirmed hot zones of 200–1000 nm, which had heated by at least 207 K, to melt the coating. Based on this observation, an initial core temperature of 3400–8600 K was estimated, depending on assumed deformation time. The same technique was later applied by other groups, but molten coating was in those cases only observed on the shear band involved in final fracture^16,20^.

Several groups have tried to estimate temperature increases by assuming that work done by elastic contraction of the machine-sample system during yield is released as heat into the shear band. Wang *et al*. calculated increases of up to 1600 K, with hot zones in the range of 1 µm reaching 500 K increase^26^. Similar studies estimated up to 2000 K in a very narrow band, resulting in a 450 K increase in a 0.4 µm hot zone, with the additional note that heating was heavily dependent on assumed shear time, becoming negligible at around 100 ms and upwards^19^. Finite element simulations have also showed that the temperature rise in a shear band can reach values higher than 1000 K^27,28^.

The existence of a critical shear time, distance or velocity, all intrinsically related, in relation to heating has similarly been discussed by several groups: Jiang *et al*. found a direct link between global strain rate and temperature increase in individual shear bands, using infrared thermography during compression testing^29^. Other groups found critical shear step sizes^18^ and sliding velocities^25,30^ for a shear band to destabilize, relating these factors to viscosity drop caused either by temperature or shear strain rate.

Aside from theoretical approaches, the only method allowing direct measurements of the timescale of events has been high frame rate thermography. Such studies have typically given temperature increase values over the duration of shear events in the range 1 to 6 K^22,29,31^. This technique is however, despite rapidly advancing technology, still limited in spatial and temporal resolution to observing hot zones in the 10 µm range after heat has dissipated from the shear band.

Testing and application of metallic glasses at cryogenic temperatures has some important benefits. Strength and plasticity have both been found to increase^11–13^. Further, some groups report the concentration of plastic deformation into few, or even single, shear bands, as opposed to the normally expected multitude^10,13^. The combination of increased plasticity and localization of deformation significantly facilitate the study of shear band dynamics at reduced temperatures.

Here, using a novel “nitrogen bubbles heat sensor” technique, we resolve the shear band dynamics of a tensile specimen submerged in liquid nitrogen (LN2) into three separate stages: steady-state shear, runaway shear and fracture. We study the timescale and related temperature evolution of each step by combining *in-situ* measurements of the stress-strain relationship and high frame rate imaging of deformation and nitrogen boiling on the deforming sample surface, with post-mortem analysis through Atomic Force Microscopy (AFM) and Scanning Electron Microscopy (SEM).

## Results

### *In-situ* tensile deformation analysis

**Figure 1:**
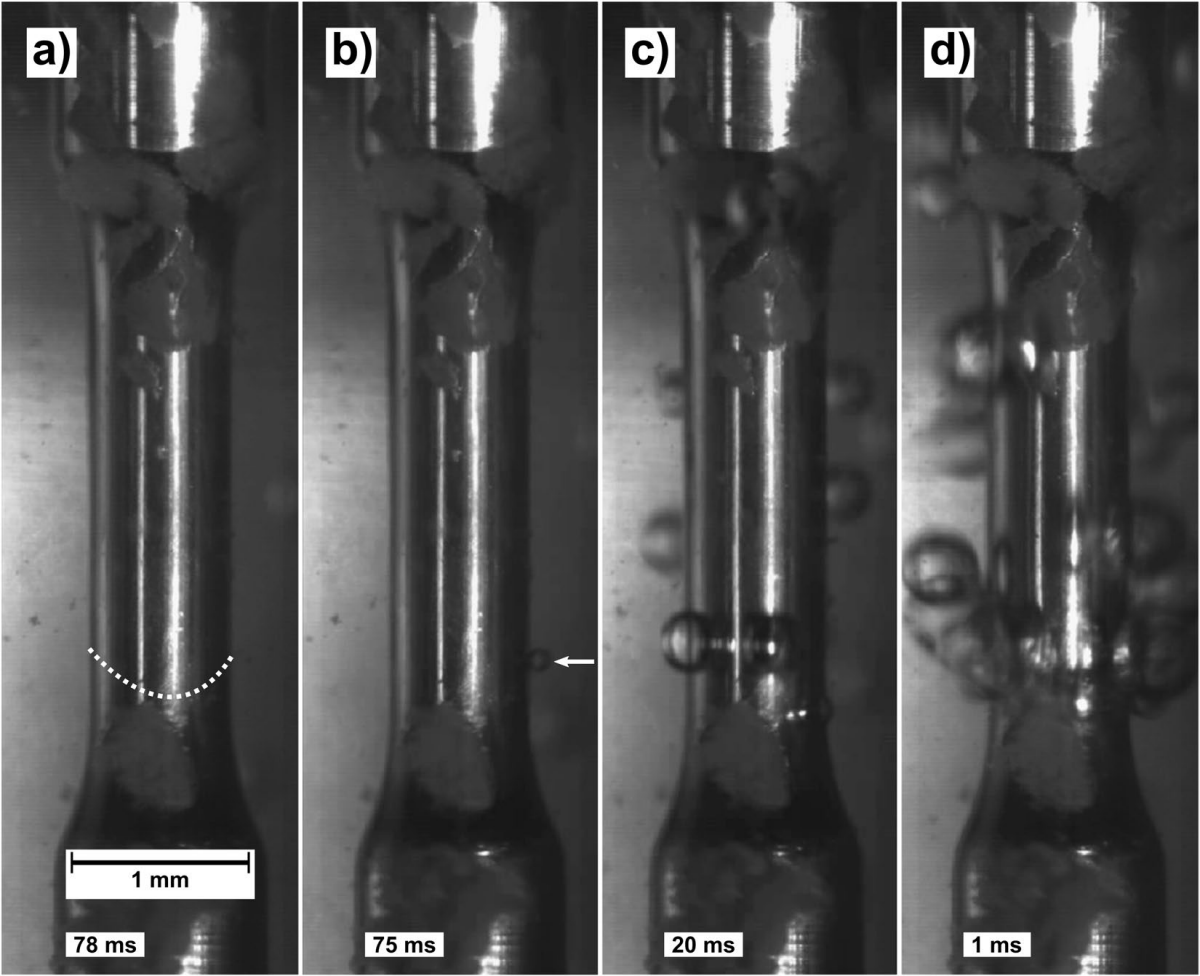
Video snapshots at various stages of plastic deformation during uniaxial tensile loading while submerged in a LN2 bath: (**a**) The sample is shown at 78 ms before fracture (after 0.03% plastic deformation) with no apparent nitrogen gas bubble nucleation. The dotted white line indicates the primary shear plane, along which fracture will eventually occur. (**b**) The sample is shown at 75 ms before fracture (after 0.035% plastic deformation) when the first bubble can be seen nucleating, marked by a white arrow. (**c**) At 20 ms (after 0.09% plastic deformation), bubble nucleation has started accelerating towards violent boiling. (**d**) At 1 ms before fracture (after 0.11% plastic deformation), violent boiling of the LN2 along the primary shear plane is apparent. The arrows in Fig. 2 correspond to the location of each of the snapshots.

We investigated the tensile response of a Zr_70_Ni_16_Cu_6_Al_8_ Bulk Metallic Glass (BMG) at a nominal strain rate of 10^−2^ s^−1^ (effective strain rate measured to 2*10^−3^ s^−1^ during elastic deformation, due to machine compliance). The testing was performed at cryogenic temperature (77 K) with the sample and part of the testing rig submerged in a LN2 bath. A high frame rate camera was used to record the specimen response during testing. Importantly, we observed nitrogen vapor formation on the sample surface during the latter stages of deformation, as direct evidence of heat release. Figure 1 shows a series of selected video snapshots (see also supporting video) at various degrees of sample deformation and surface boiling.

**Figure 2:**
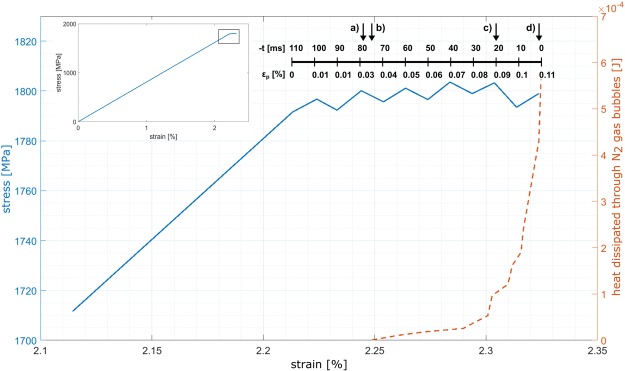
Plastic part of engineering stress-strain relationship (blue line) overlaid with corresponding cumulative energy expenditure for gas evaporation (dashed red line). The inset at the upper left hand corner shows the full stress-strain relationship. The bar above the plastic part of deformation shows time before fracture in ms as well as plastic deformation, with the arrows marked (a) through (d) indicating the locations that correspond to the snapshots in Fig. 1.

**Figure 3:**
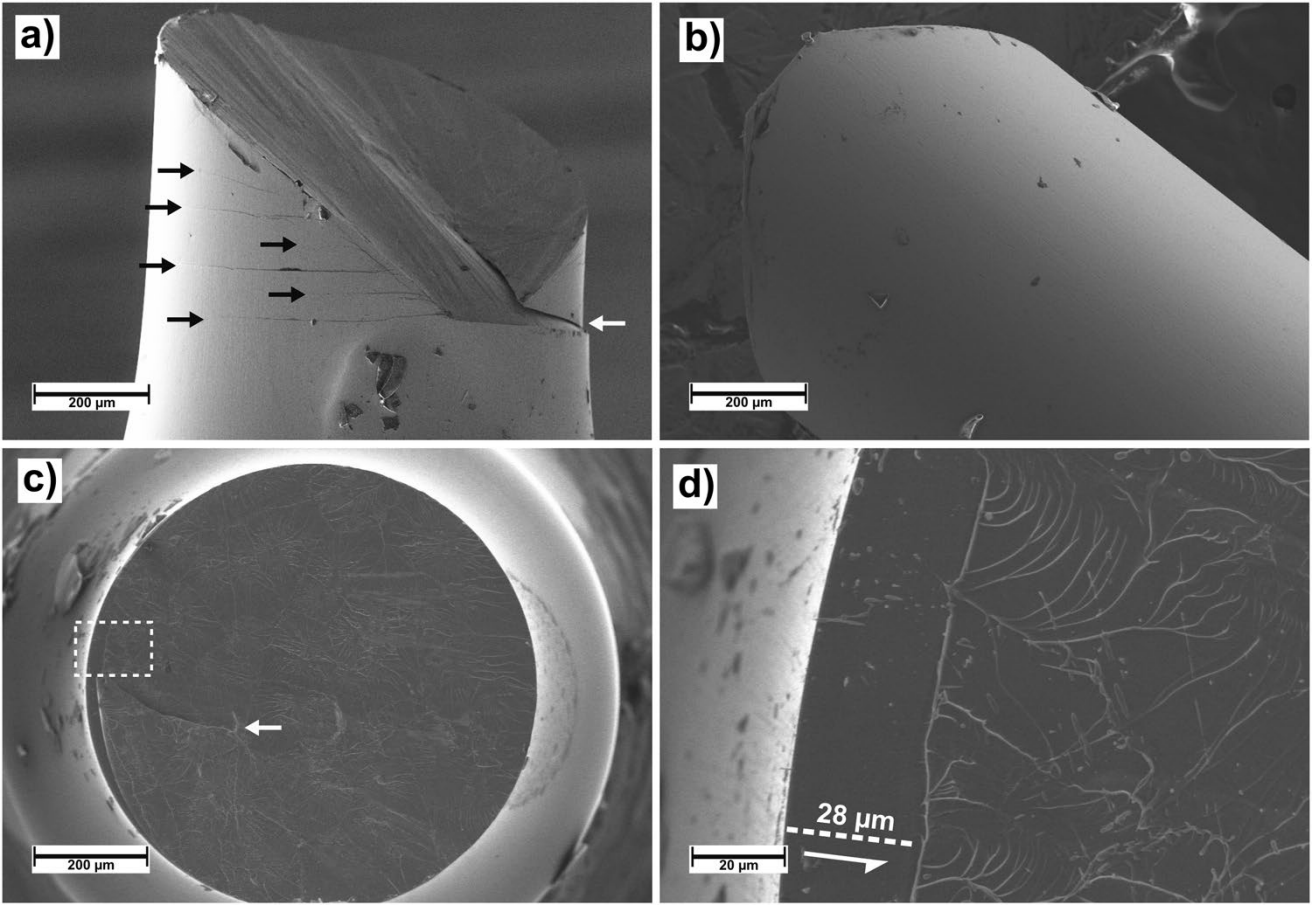
SEM micrographs of tensile specimen after fracture at cryogenic temperature. Several secondary shear bands, marked by black arrows in (**a**), can be seen emerging from the primary shear band. The secondary shear bands do not continue to the other half of the sample, (**b**), indicating they were generated after the primary. Overview of the fracture surface, (**c**), reveals a featureless sliding zone, while the remaining surface is covered in characteristic Saffman-Taylor fingering. In (**d**), the dotted square in (**c**) is enlarged to measure the sliding zone length. Shear direction is indicated by a white arrow. A crack in the surface, marked by white arrows in (**a**,**c**), indicates that sliding first happened along the primary shear band, before final fracture occurred along the intersection of the primary and a secondary shear band. The dark particles on the surface, primarily visible in (**a**,**b**), are remainders of tracer markings used for image analysis^13^.

Figure 2 shows an enlarged view of the plastic deformation part of the recorded stress-strain relationship, with the full curve as an inset. The upper horizontal axis in the figure gives the corresponding timeline, in ms before fracture occurs, as well as the plastic deformation. The sample undergoes about 2.2% elastic deformation, followed by a relatively limited 0.11% plastic response. SEM images of the two sides of the fractured sample, (Fig. 3a,b), indicate that the plastic deformation was mainly accommodated along the primary shear band. This high degree of localization is in accordance with previous reports for cryogenic tensile testing of BMG^10,12,13,32^, and will be discussed in further detail later.

Let us now discuss the nitrogen gas bubbles forming on the sample surface during plastic deformation. It is interesting to note that no boiling was observed on the sample surface during the elastic regime, see (Fig. 1a). The first nucleated bubble, at 0.035% plastic deformation, can be seen at 75 ms before fracture, marked by the arrow in (Fig. 1b). After relatively stable boiling for 40 ms, the formation rate of bubbles increases drastically, as in (Fig. 1c), resulting in violent boiling of the LN2 bath in the final ms before fracture occurred, shown in (Fig. 1d). We also note that all bubbles were generated along the same plane, as marked by the dotted line in (Fig. 1a). This line corresponds to the primary shear band that later results in fracture as seen in (Fig. 3a,b). The bubbles seen above this line in (Fig. 1c,d) have risen due to buoyancy in the LN2 bath (Supplementary Video).

Boiling incipience below the homogeneous nucleation temperature of LN2 (105 K)^33^, is driven by both the wall and bath superheat as well as the favorability of nucleation sites^34,35^. The presence of nucleating sites along the entire shear band, considering that the shear step geometry varies along its boundary, as well as the fact that the bath was kept open to the atmosphere, indicates that wall superheat is the main driving factor for bubble formation. Thus, the above observations can be considered as direct evidence of heating in the shear band.

In what follows, we attempt to quantify the heat necessary for the formation of the observed bubbles. The work done by bubble growth consists of enthalpy of vaporization, formation of new interfacial surface and displacement of a liquid volume by gas^34^. The latter two are negligible for bubbles of the observed size, so we focus on the former.

Studying the recorded video frame-by-frame, we measure the time of nucleation as well as the time and diameter at release from the sample surface for each bubble. We then use the ideal gas law with the volume of a sphere inserted to estimate the number of evaporated LN2 molecules, *n*, by

1 $$n=\frac{4P\pi {r}^{3}}{3RT}$$

where *P* is internal gas pressure (assumed atmospheric), *r* bubble radius, *R* the universal gas constant and *T* is temperature (approximated here to the equilibrium 77 K). The number of gas molecules can then be directly translated to consumed heat by the heat of vaporization, ${\rm{\Delta }}{H}_{vap}=5.57\,kJ/mol$ for nitrogen^36^. A total heat consumption of 5.6*10^−4^ J is measured by the final frame, with the whole estimated heat profile plotted by the red, dashed line in Fig. 2.

N_2_ close to its boiling point does not act as a perfectly ideal gas, so the use of Equation 1 must be regarded as an approximation. However, the trend of heat dissipation from the shear band through the LN2, as shown in Fig. 2, should be independent of the actual deviation from ideal behaviour.

Released heat from the shear plane increases in a stable, quasi-linear fashion between 0.035% and 0.08% plastic strain, as seen in Fig. 2. The heat release starts to increase drastically at about 0.09% of plastic strain, Fig. 2, indicating an apparent change in the shear band propagation. We consider the deformation recorded on the stress-strain curve as part of the steady state regime^7^, as the shear propagation rate seems to be approximately constant and relatively slow. The change however in boiling dynamics foreshows the transition into “runaway” shear^30^, as we observe both increased heating and a slight apparent stress drop. This transition will be discussed in further detail later. During steady-state flow, effective strain rate on the sample is equal to the nominal strain rate 10^−2^ s^−1^, while the stress drop during runaway shear gives an increase in strain rate, due to a simultaneous elastic relaxation of the machine-sample system. The acceleration of shear and temperature increase relate to a lowered viscosity in the shear banding runaway regime^7,18,30^, eventually leading to imminent fracture.

### Post-mortem analysis

To gain further insight into the late stages of deformation and fracture, we investigate the post-mortem fracture surface top-down using SEM. In (Fig. 3c) we see an overview of the surface mostly covered in the characteristic Saffman-Taylor fingering indicating separation along a viscous layer between two plates^8,9^. Along the left-hand edge, a smooth, featureless sliding zone, evidence of shear sliding before final fracture can be seen. A crack, also observed in (Fig. 3a), indicates that final fracture occurred along the intersection of the primary shear band and a major secondary shear band. Generation of the secondary shear band happened after sliding of the primary, evident by the lack of a sliding zone above the crack.

The featureless zone seen in (Fig. 3c), is studied in further detail in (Fig. 3d). The widest part of the featureless zone indicates the shear displacement of the two surfaces before fracture, and is measured to 28 µm in the top-down projection. Accounting for the 54 ° shear band angle to the tensile axis in the present sample, this measurement gives a total in-plane shear displacement of 34.6 µm. Interestingly, the 0.11% plasticity in the stress-strain curve only corresponds to 4.3 µm in-plane sliding, marking a clear discrepancy between *in-situ* and post-mortem observations.

We therefore divide shear deformation into two regimes by what is observable by the testing rig, *i.e*. 4.3 µm steady-state and 30.3 µm runaway shear. Comparing the timescales of the events, the steady-state shear happened over 110 ms, while the runaway sliding occurred in less than 1 ms, as it was neither caught by the testing rig (temporal resolution 100 Hz) nor the camera (spatiotemporal resolution of 6.4 µm/px and 1000 Hz). We can use these numbers to calculate propagation velocities in the respective regimes: For the steady-state shear, we assume constant velocity^23^ directly translatable from the cross-beam displacement, as there was no sufficient stress drop to make system compliance significant. The measured strain rate gives an in-plane sliding velocity of 4*10^−5^ ms^−1^. In comparison, the runaway shear happened at an average velocity of at least 3*10^−2^ ms^−1^. There is hence at least a three order of magnitude difference in velocities.

Although supercooled metallic liquids are not fully Newtonian^37^, we approximate shear layer viscosity between two plates, η, by

2 $$\eta =\tau \frac{h}{v}$$

where *τ* is the shear stress, *h* the viscous layer thickness and *v* the sliding velocity^23^. For *h* we use 20 nm, which corresponds to an estimated initial shear band thickness^6^. As the area reduction at the end of the shear event was only ~5%, we simplify *τ* to

3 $$\tau ={\sigma }_{y}\,\sin \,\alpha \,\cos \,\alpha $$

where *σ*_*y*_ is yield stress 1790 MPa and *α* the shear band angle 54°. Viscosities for the two cases are then calculated to 4.4*10^5^ Pas and 5.6*10^2^ Pas for steady-state and runaway shear, respectively.

In the case of steady-state shear, the calculated viscosity is presumably a slight under-estimate, as the viscous liquid layer thickness might grow throughout the duration of deformation. In the runaway case, the calculated viscosity is an upper bound. Real velocity can be orders of magnitude higher than the minimum we estimate and there is most likely also a significant shear stress drop, which would both decrease viscosity. Potential liquid layer thickness increase is limited in its contribution to a maximum factor of ~6, as final thickness after fracture is 120 ± 40 nm, found through volumetric integration of surface features measured by AFM. In the equilibrium state, the calculated viscosities would correspond to temperatures of 743 K and 908 K for steady-state and runaway shear, respectively, both well above the 630 K glass forming temperature of the alloy^38^. These figures are, however, not directly translatable, considering the viscosity dependence on stress state, heating rate and temperature^7,37,39^.

Using the findings on stress and displacement, we quantify the heat generation inside the shear band. Work done by shear deformation is found by

4 $$W=\tau \delta $$

where *δ* is the shear offset^40^. Not accounting for energy consumed by structural changes, which should be negligible for displacements of the observed magnitude^18^, we treat this work as equal to heat released during the shear banding event, H. For steady-state shear, where we know that *σ* = *σ*_*y*_, *H* becomes 3.7 kJm^−2^, for a total of 2.3*10^−3^ J over the shear band area. Interestingly, this value is about 4 times higher than the heat consumed by the bubble formation in the steady state shear regime (5.6*10^−4^ J, (Fig. 2) implying that about 25% of the heat generated by the work done was dissipated to the LN2 bath through the shear plane. Due to a stress drop of unknown magnitude, the subsequent runaway shear generated less than, but probably close to, 23 kJm^−2^, 1.44*10^−2^ J in total.

### Fracture

**Figure 4:**
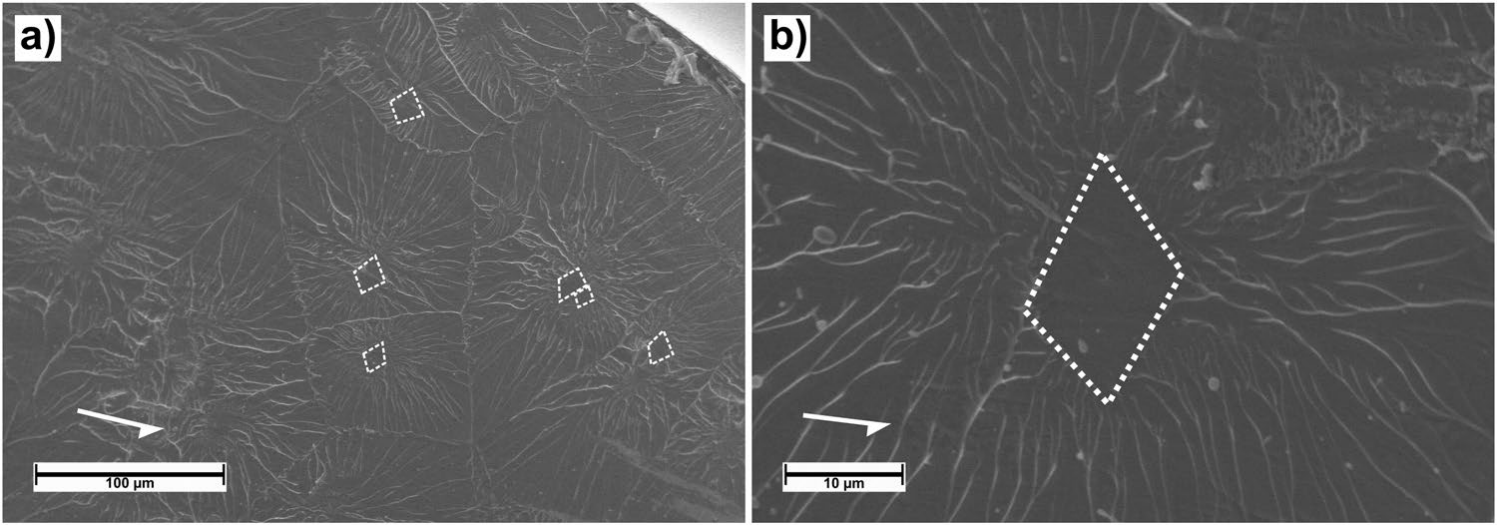
Fracture morphology showing the characteristic viscous fracture. (**a**) Overview micrograph of fracture surface showing Saffman-Taylor fingering radiating out from smooth cores. Some distinctly rhombus-shaped cores are marked by dashed lines. (**b**) Shows a magnified image of a rhombus-shaped core, with the arrow marking shear band sliding direction.

Studying the fracture morphology of the sample in more detail, we obtain information on the event progression during the catastrophic failure itself. Figure 4 shows some important fracture features. The segregation of fracture domains, marked by smooth cores and radiating veins, is distinct on a large part of the surface at 77 K, in accordance with previous reports on typical tensile fracture surfaces^11,41,42^. The nucleation cores are marked in (Fig. 4a) by dashed lines. Some of the highly localized cores are approximately rhombic in shape, as seen more closely in (Fig. 4b), and align with each other and roughly with the shear direction.

The ridges demarking the domains of their respective voids forming a Voronoi tessellation implies that fracture has initiated at each of these voids simultaneously. The lack of directionality in the vein pattern further indicates that the present fracture has been driven outwards by normal stresses, rather than shear. Several of the nucleation cores are rhombus-shaped, oriented along the direction of deformation. This shape suggests involvement of shear forces in the core nucleation, fitting well with shear induced cavitation^30^ in the late stages of failure.

The presence of a viscous ridge defining the featureless zone, as seen in (Fig. 3d), further suggests that final separation has not initiated along this line and originates mainly from the cores.

## Discussion

**Figure 5:**
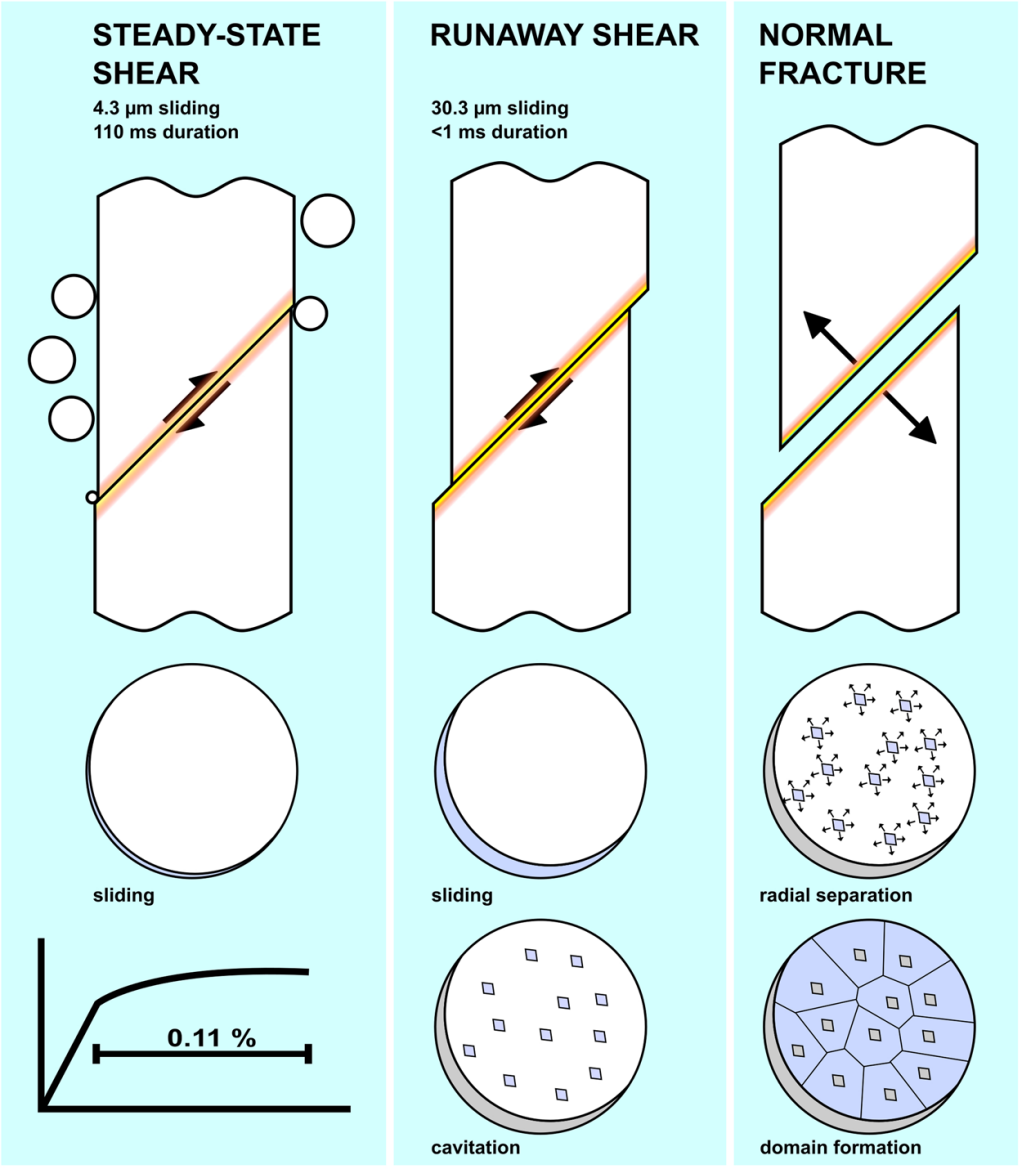
Side and top-down view of the progressive stages of deformation and failure. Active stages in the top-down view are marked in deep blue, while previous stages are grey. First, we observe limited steady-state deformation, with bubbles nucleating along the shear band edges. Further larger scale sliding happens in the runaway state, with increased heat release, ending in shear induced cavitation. The final fracture spreads outwards from the shear induced cores.

Based on the combination of *in-situ* and post-mortem analysis, we estimate the deformation-and-fracture sequence as summarized in Fig. 5. Steady-state shear happened over the duration of 110 ms, comprising 0.11% plastic deformation. In the latter 77 ms of this deformation stage, we observe an increasing number of bubbles nucleating on the sample surface. The incipience of boiling is 33 ms after onset of plasticity, and the heat release remains relatively low, ~0.5*10^−4^ J up to 0.085% of measured steady-state strain. In the final 0.025% of observable plastic strain, the released heat increases by an order of magnitude, and presumably further during runaway shear not captured in Fig. 2. Thus the results point towards the heat release following the operation of the shear band, in accord with previous theoretical analyses by Jiang *et al*.^43^, as well as experimental results^16,20^.

If we assume an initial hot zone around the shear band of 1 µm, the wall heat flux during the initial stable boiling becomes ~0.3 MWm^−2^. Comparing this to previous experiments by Okuyama and Iida, this flux should result in boiling incipience at a wall superheat around 20 K^33^. The required wall superheat could however be somewhat lower if the surrounding LN2 were already superheated. The temperature at the end of shear could approach 1000 K, based on the equilibrium viscosity calculations.

Using Equation 1, we calculate a total heat consumption of 5.6*10^−4^ J, equivalent to 25% of the work done, *i.e*. 2.3*10^−3^ J as calculated by Equation 4. The consumed heat cools the sample surface, so that no new bubble nucleates until sufficient superheat is provided again^44^, explaining why a single spot does not continue to nucleate bubbles immediately after being activated. The increase in nucleation rate towards the end of steady-state shear premonitions the transition into runaway deformation.

Sun *et al*.^30^ proposed 10^−4^-10^−3^ ms^−1^ as a threshold between steady-state and runaway shear. The velocities calculated for the present sample are 4*10^−5^ and at least 3*10^−2^ ms^−1^ for the two regimes respectively, falling neatly on either side of that threshold. The latter could, however, be several orders of magnitude higher. Correspondingly, previously reported values suggest above 10^−3^ ms^−1^ for hot shear bands in similar glasses^18^.

The transition from runaway shear to final fracture occurs once the viscous layer is sufficiently unstable to allow shear induced cavitation^30^. In (Fig. 4a,b) we observe Saffman-Taylor fingering^8,9^ radiating out from smooth cores. This fracture morphology is similar to that seen in previous reports on tensile testing, at both room^32^ and cryogenic temperature^11,42,45^. Some previously reported surfaces show round cores with a degree of directionality in the viscous fingering, consistent with the shear direction^11,32^, indicating normal stress driven cavitation and shear driven final fracture. However, our results show several cavitation cores with clearly defined rhombic shapes, roughly oriented in the shear direction, as in (Fig. 4b). This morphology indicates involvement of shear forces during cavitation. That the veins radiate omnidirectionally and form a Voronoi tessellation, see (Fig. 4a), is indicative of normal stress domination when driving the voids outwards. Previously, it has been suggested that the cores themselves nucleate due to normal stresses, but are then driven outward by shear^11,32^. Our results seemingly show the opposite order of dominating stresses.

## Conclusion

We observe steady-state shear band propagation during tensile testing at 10^−2^ s^−1^ in LN2 through 0.11% macroscopic plasticity. Heat generation is evidenced at this stage by local boiling of the surrounding LN2 bath. The boiling consumes an estimated 5.6*10^−4^ J, corresponding to about 25% of the calculated work done by shear deformation. Steady-state shear occurred over 110 ms, with an acceleration in boiling marking the imminent destabilization of the band. The commencement of boiling after 0.085% plastic strain, and at a relatively low wall superheat, indicates that the heating follows the operation of the shear band. Once the shear band reaches runaway instability, its propagation accelerates by several orders of magnitude, and can only be observed post-mortem. At a critical point, the liquid layer is sufficiently weakened to let shear forces nucleate fracture through cavitation at several sites across the shear band simultaneously. These voids spread outwards by normal forces, generating a surface covered in the characteristic Saffman-Taylor fingers radiating outwards from each void.

The presented findings give a novel way of analyzing heating and propagation of shear bands. The clear observation of the steady-state shear band dynamics helps pinpoint the limit below which the deformation is still arrestable, and the bulk metallic glass can be safely used. This approach could be translated directly to ambient temperature cases by submerging the sample in various liquid mixtures with different boiling points and enthalpies of evaporation.

### Experimental Procedures

Zr_70_Ni_16_Cu_6_Al_8_(ZNCA) master alloy ingots were produced by arc melting high purity elements in a Diavac Limited custom-made, automatic arc melter. A Nissin Giken double torch arc melter was used to produce ø = 3.5 mm, l = 12.5 mm cylindrical rods by tilt casting^46^. Full vitrification was ensured by X-ray diffraction using a Rigaku Ultima IV with a Cu target and 20° < 2θ < 100°. Tensile specimens with gauge area ø = 0.8 mm and l = 2.3 mm were machined from the rods. P4000 lapping tape was used to polish the specimen gauge areas, which were inspected by optical microscopy.

A specially made sample holder consisting of two coaxial rods allowed the tensile specimen to be submerged in LN2 while mounted in a displacement controlled Shimadzu AG-X tensile testing machine with a data gathering frequency of 100 Hz, and a resolution of 15 kPa and 20.8 nm. A high frame rate camera, Photron FASTCAM SA1.12, was used to record the sample through a window in the LN2 container during deformation. The sample was tested at nominal strain rate 10^−2^ s^−1^, with corresponding video frame rate 1000 Hz and a resolution of 6.4 µm per pixel. The actual strain rate during elastic deformation in the sample was 2*10^−3^, due to low machine stiffness resulting from the required sample holder setup. Digital image correlation was used to find the stress-strain relationship, as explained elsewhere^13^. Note that the plotted irregularities in the plastic deformation correspond in magnitude to load cell resolution, and do not necessarily reflect real serration events like observed in compression testing^19^. The elastic region was smoothed mathematically, while the plastic region was left unsmoothed to preserve more accurate data. The present work focuses on a single sample which is a subset of a previously reported work^13^. The reported boiling was, however, observed for all samples tested in LN2. Only one sample was chosen for deeper analysis due to its coincidence of plastic deformability, high video quality and a suitable strain- and frame rate.

Kinovea 0.8.15 software was used to study the resulting video frame-by-frame. The diameter of bubbles evolving along the shear band was measured at the frame of release. Timestamps for first appearance and release from the sample surface were noted. Bubbles hidden behind the sample were accounted for by a correction factor of 17%, based on average bubble size compared to the sample width. A low angle of view limited size distortion of bubbles, allowing direct measurements regardless of position relative to the specimen.

SEM was used to inspect the sample side and fracture surface. A Zeiss Ultra 55 and a Hitachi S−3400N were used, both in secondary electron mode.

**Figure 6:**
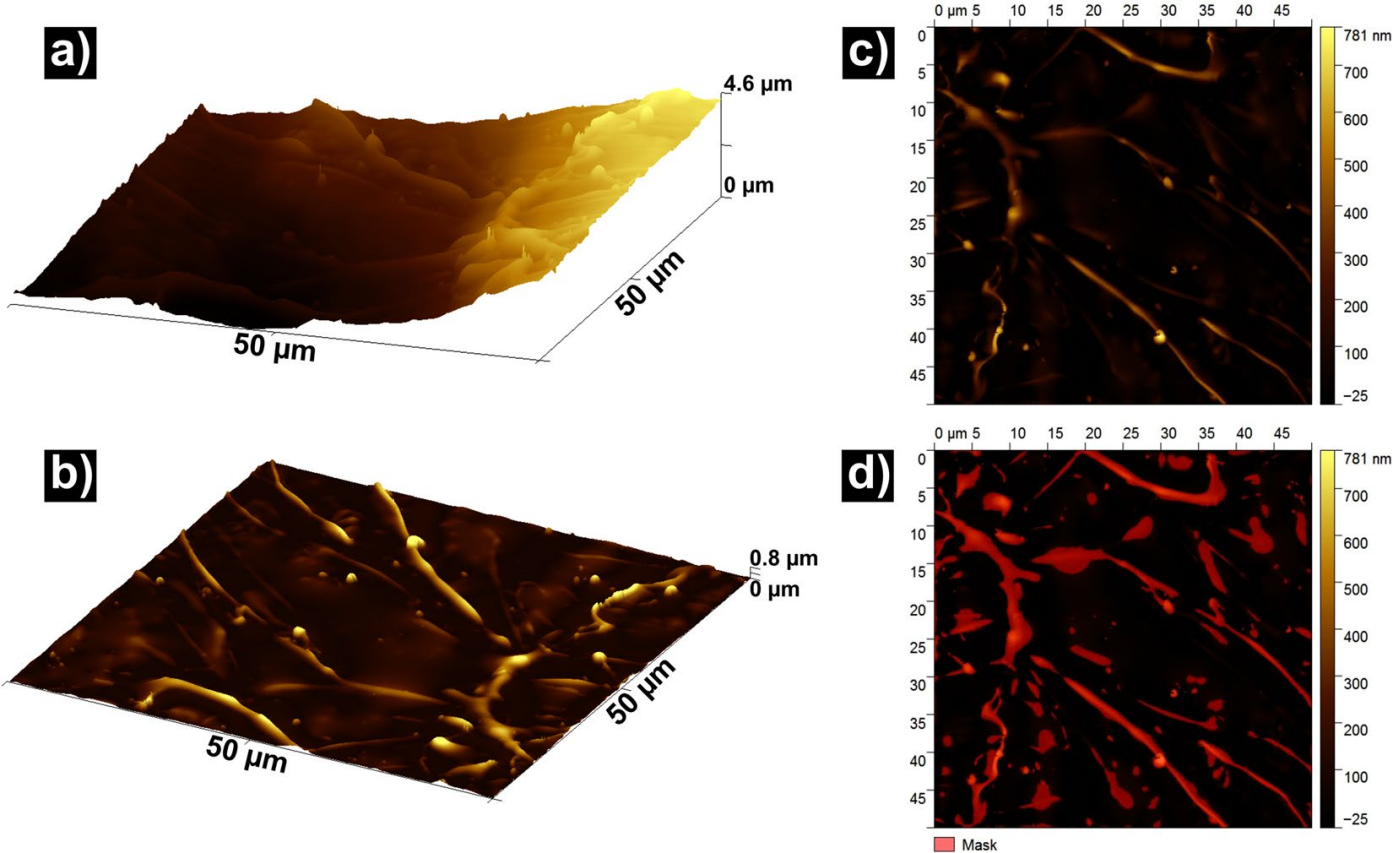
AFM scan of a selected area on the fracture surface, detailing the process of the viscous layer thickness measurement. (**a**) Shows the original scan in 3D, while (**b**) is the same area after flattening through polynomial subtraction. (**c**) is a map of the same area, where (**d**) is a mask of the highest 80%, considered to constitute the viscous layer involved in shear and fracture.

The thickness of the viscous layer was measured by studying the fracture surface by AFM, in an Agilent 5500 microscope. 9 representative 50 × 50 µm^2^ spots across the sample were scanned. Results were visualized and analyzed using Gwyddion 2.49 software^47^. The total volume of the present vein pattern was found by first subtracting the global topography of the scan by an automatically generated 11th degree polynomial (highest possible in the program). The highest remaining part (80%) of the sample surface was then taken to represent the Saffman-Taylor fingering^9^, which was spatially integrated using an automatic feature in the software. The calculated volume was then used to find the thickness of the original layer. The stepwise procedure for an example scan is shown in Fig. 6 where (a) depicts the initial scanned area, (b) and (c) the same area after polynomial flattening and d) shows the raised areas taken to have composed the liquid layer, which is then spatially integrated.

## Electronic supplementary material

Shear band surface boiling
